# Redirection of the central metabolism of *Klebsiella pneumoniae* towards dihydroxyacetone production

**DOI:** 10.1186/s12934-021-01608-0

**Published:** 2021-06-29

**Authors:** Shaoqi Sun, Yike Wang, Lin Shu, Xiyang Lu, Qinghui Wang, Chenguang Zhu, Jiping Shi, Gary J. Lye, Frank Baganz, Jian Hao

**Affiliations:** 1 Lab of Biorefinery, Shanghai Advanced Research Institute, Chinese Academy of Sciences, No. 99 Haike Road, Pudong, Shanghai, 201210 People’s Republic of China; 2 Department of Biochemical Engineering, University College London, Gordon Street, London, WC1H 0AH UK; 3 School of Life Science, Shanghai University, Shanghai, 200444 People’s Republic of China; 4 University of Chinese Academy of Sciences, Beijing, 100049 People’s Republic of China; 5 School of Life Science and Technology, ShanghaiTech University, Shanghai, China

**Keywords:** Dihydroxyacetone, Glycerol, *tpiA*, *hdpA*, *Klebsiella pneumoniae*

## Abstract

**Background:**

*Klebsiella pneumoniae* is a bacterium that can be used as producer for numerous chemicals. Glycerol can be catabolised by *K. pneumoniae* and dihydroxyacetone is an intermediate of this catabolism pathway. Here dihydroxyacetone and glycerol were produced from glucose by this bacterium based a redirected glycerol catabolism pathway.

**Results:**

*tpiA*, encoding triosephosphate isomerase, was knocked out to block the further catabolism of dihydroxyacetone phosphate in the glycolysis. After overexpression of a *Corynebacterium glutamicum* dihydroxyacetone phosphate dephosphorylase (*hdpA*), the engineered strain produced remarkable levels of dihydroxyacetone (7.0 g/L) and glycerol (2.5 g/L) from glucose. Further increase in product formation were obtained by knocking out *gapA* encoding an iosenzyme of glyceraldehyde 3-phosphate dehydrogenase. There are two dihydroxyacetone kinases in *K. pneumoniae*. They were both disrupted to prevent an inefficient reaction cycle between dihydroxyacetone phosphate and dihydroxyacetone, and the resulting strains had a distinct improvement in dihydroxyacetone and glycerol production. pH 6.0 and low air supplement were identified as the optimal conditions for dihydroxyacetone and glycerol production by *K, pneumoniae* Δ*tpiA-*Δ*DHAK-hdpA*. In fed batch fermentation 23.9 g/L of dihydroxyacetone and 10.8 g/L of glycerol were produced after 91 h of cultivation, with the total conversion ratio of 0.97 mol/mol glucose.

**Conclusions:**

This study provides a novel and highly efficient way of dihydroxyacetone and glycerol production from glucose.

**Supplementary Information:**

The online version contains supplementary material available at 10.1186/s12934-021-01608-0.

## Background

*Klebsiella pneumoniae* is a facultative anaerobic gram-negative rod-shaped bacterium and belongs to the family of *Enterobacteriaceae*. It is ubiquitous distributed in natural environments and can catabolise many kinds of carbon sources. *K. pneumoniae* is also an important industrial microorganism. Wild type strains have been used as producers for 1,3-propanediol and 2,3-butanediol production. After metabolic engineering modification, *K. pneumoniae* strains have been used as workhorses for 2-ketogluconic acid [1], gluconic acid [2], xylonic acid [3], acetoin [4], 2,3-dihydroxyisovalerate [5], 2-ketoisovalerate and isobutanol production [6].

Glycerol is a suitable carbon source for *K. pneumoniae* growth, and it was used for 1,3-propanediol production. The glycerol catabolism pathway is a dismutation process. In the oxidative branch, glycerol is oxidized to dihydroxyacetone (DHA) under the catalysis of glycerol dehydrogenase, and the latter is then phosphorylated to DHA phosphate and channeled into glycolysis. In a coupled reductive pathway, glycerol is converted to 3-hydroxypropionaldehyde and further converted to 1,3-propanediol [7]. Glycerol dehydrogenase, glycerol dehydrase and 1,3-propanediol dehydrogenase are encoded by *dhaD*, *dhaB* and *dhaT*, respectively. There are two DHA kinases in *K. pneumoniae*. DHA kinase I use ATP as the cofactor and DHA kinase II use phosphoenolpyruvate (PEP) as the cofactor [8]. DHA kinase I is encoded by *dhaK*. DHA kinase II consists of three subunits, and they are encoded by *dhaK1*, *dhaK2* and *dhaK3*, respectively. Genes of those enzymes are in an operon named *dha* operon [9]. When glycerol is used as the carbon source, metabolites of *K. pneumoniae* are 1,3-propanediol, 2,3-butanediol and some organic acids [7].

DHA is a simple, achiral, and non-toxic sugar. It is used extensively in the cosmetic industry for making artificial suntans. DHA is used as a building block for the synthesis of lactic acid and 1,2-propanediol. DHA is also extensively used in the medical industry [10]. DHA can be synthesized through a chemical route with formaldehyde as the substrate, but the product is a mixture of many unbranched aldoses and ketoses [11]. DHA was efficiently produced from glycerol through a biotransformation process by *Gluconobacter oxydans*. In *G. oxydans*, the enzyme responsible for the oxidation of glycerol to DHA is a membrane-bound glycerol dehydrogenase, which use pyrrolequinone as the cofactor [12]. A *Pichia membranifaciens* strain was reported for the production of DHA from glycerol, but the responsible enzymes were unidentified [13]. A mutant of *Hansenula polymorpha* was reported to produce dihydroxyacetone from methanol in a resting-cells reaction. Methanol was catabolised through a xylulose monophosphate cycle pathway in this yeast [14].

*Corynebacterium glutamicum* has a metabolic pathway that convert sugars to glycerol via DHA as an intermediate, and this pathway has been used for 1,2-propanediol and 1-propanol producing strains construction [15]. DHA phosphate is an intermediate in the glycolysis and was converted to DHA by a *hdpA* encoded DHA phosphate dephosphorylase. DHA was further converted to glycerol or converted to methylglyoxal for 1,2-propanediol production. A DHA producing *E. coli* strain was constructed through expression of the *G*. *glutamicum hdpA* gene [16].

**Fig. 1:**
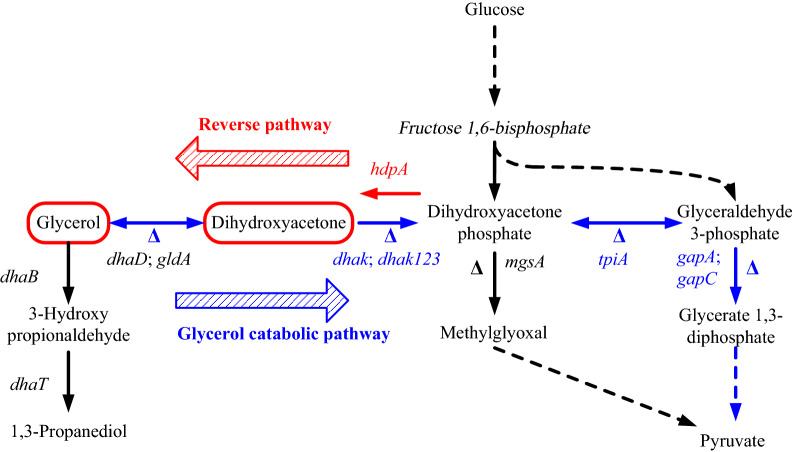
Glycerol catabolic pathway, redirected pathway, and related pathways of *K. pneumoniae*

DHA is an intermediate of the glycerol catabolism pathway in *K. pneumoniae*. But it cannot accumulate in the process. Cultivation of *K. pneumoniae* that use glucose or other sugars as the carbon source, produce as main metabolites 2,3-butanediol and some organic acids [17], glycerol or DHA were not metabolic products of this process. In this work, redirection of glycerol catabolic pathway was done in *K. pneumoniae* for DHA and glycerol production from glucose (Fig. 1).

## Results

### Thermodynamic analysis of glycerol catabolic pathway

Gibbs free energy (ΔrG) and equilibrium constant (K_eq_) of reactions and pathways related to glycerol catabolism were estimated with the help of eQuilibrator, and results are given in Table 1.

**Table 1:** Gibbs free energy for reactions

No.	Reactions and pathways	Enzyme	ΔrG (kJ/mol)	K_eq_
1	Glycerol + NAD^+^ = DHA + NADH + H^+^	DhaD	24.6 ± 3.6	4.9 × 10^–5^
2	DHA + ATP = DHA-P + ADP	Dhak	− 13.7 ± 4.3	255
3	DHA + PEP = DHA-P + pyruvate	Dhak123	− 41.4 ± 4.4	1.8 × 10^7^
4	DHA-P + H_2_O = DHA + phosphate	HdpA	− 12.7 ± 4.3	166
1 + 2	ATP + NAD^+^ + Glycerol = NADH + ADP + DHA-P	DhaD, Dhak	10.9	
1 + 3	PEP + NAD^+^ + Glycerol = NADH + pyruvate + DHA-P	DhaD, Dhak123	− 16.8	
4–1	NADH + DHA-P = NAD^+^ + Glycerol + phosphate	DhaD, HdpA	− 37.3	

DhaD catalyses the conversion of glycerol to DHA, and NAD is used as the cofactor (No. 1 in Table 1). The estimated ΔrG of this reaction was positive, and K_eq_ was lower than 1. These results indicated that this reaction prefers glycerol formation rather than DHA formation. At reaction equilibrium condition, the level of DHA would be much lower than that of glycerol.

DHA kinase I that is encoded by *dhaK* catalyses DHA phosphate (DHA-P) formation and this reaction use ATP as the phosphate donor (No. 2 in Table 1). The estimated ΔrG of this reaction was − 13.7 ± 4.3 kJ/mol. Combining this reaction with the reaction of DHA formation from glycerol that is catalysed by DhaD, the ΔrG of this pathway (No. 1 + 2 in Table 1) was 10.9 kJ/mol. Consider the levels of ATP and ADP in *K. pneumoniae* were nearly equal [18]. Thus, the pathway of glycerol catabolism that is catalysed by the corresponding two enzymes was not preferred from the thermodynamic aspect. In other words, redirection of glycerol formation from DHA phosphate was possible.

DHA kinase II catalyses DHA phosphate formation with PEP as the phosphate donor (No. 3 in Table 1). The estimated ΔrG of this reaction was − 41.4 ± 4.4 kJ/mol, which was more negative than the reaction that is catalysed by DHA kinase I. Accordingly, the ΔrG of the pathway that catalysed by DHA kinase II and DhaD (No. 1 + 3 in Table 1) was − 16.8 kJ/mol. This glycerol catabolism pathway was preferred from a thermodynamic aspect.

DHA phosphate dephosphorylase (*hdpA*) of *C. glutamicum* catalyses the reaction of DHA formation from DHA phosphate (No. 4 in Table 1). DHA phosphate was hydrolyzed to release free phosphate and DHA. This reaction was not linked with ATP or PEP formation. The ΔrG of this reaction was − 12.7 ± 4.3 kJ/mol. Combining this reaction with the DhaD catalysed reaction to set up a opposite direction of glycerol catabolism pathway (No. 4–1 in Table 1), and the estimated ΔrG of this pathway was − 37.3 kJ/mol. Then, DHA and glycerol formation from DHA phosphate through this pathway was feasible from a thermodynamic aspect.

### DHA production from DHA phosphate

**Fig. 2:**
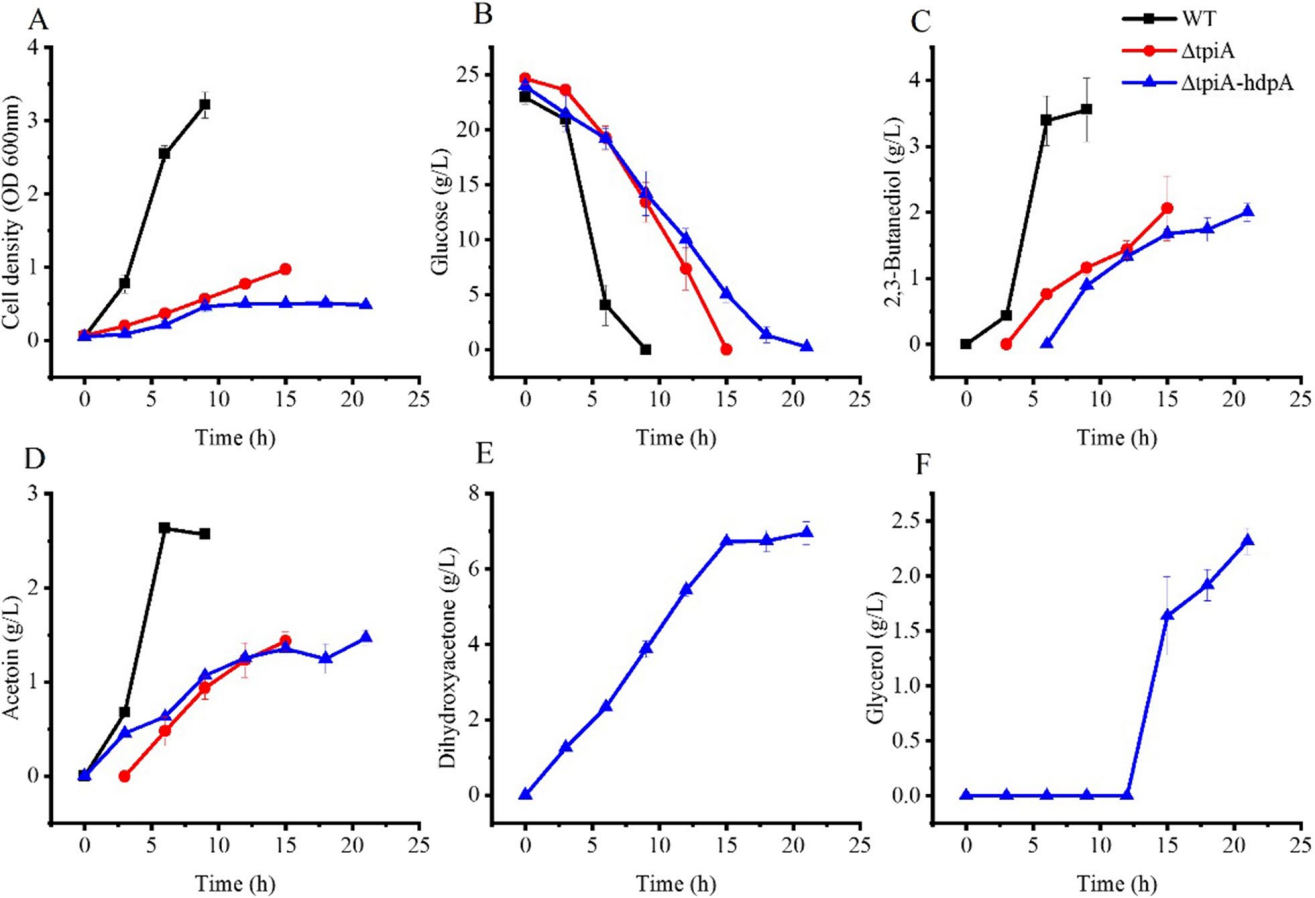
Growth and product formation of wild type *K. pneumoniae*, *Kp* Δ*tpiA* and *Kp* Δ*tpiA-hdpA* grown in shake flasks. WT: Wild-type; ΔtpiA: *Kp* Δ*tpiA*; ΔtpiA-hdpA: *Kp* Δ*tpiA-hdpA*. Data points are the average of n = 3; error bars represent standard error about the mean

The glycerol catabolism pathway that formed by DhaD and Dhak (No. 1 + 2 in Table 1) has a positive ΔrG. This indicated glycerol might be formed from DHA phosphate, which is an intermediate of the glycolysis. However, there are no reports of glycerol formation from glucose or other sugars by wild type *K. pneumoniae. Kp* Δ*tpiA* was constructed to block the conversion of DHA phosphate to glyceraldehyde 3-phosphate. This strain and the wild type of *K. pneumoniae* were cultured in flasks with glucose as the carbon source and results are shown in Fig. 2.

23 g/L glucose was consumed by the wild-type *K. pneumoniae* after 9 h of cultivation. Cell growth was coincided with glucose consumption and 3.2 ± 0.2 OD units of cell density was achieved after glucose exhausted. The main metabolites of the process were 2,3-butanediol and acetoin, and their final titers were 3.6 ± 0.5 and 2.6 ± 0.2 g/L, respectively. Low level of acetic acid and lactic acid were produced in the process (data not shown). Cell growth of *Kp* Δ*tpiA* was much slower compared with that of the wild-type strain, and the final cell density was only 1.0 ± 0.1 OD unit. This strain needed 15 h to consume all the glucose. 2,3-Butanediol and acetoin were still the main metabolites of this strain. But their titers were reduced to 2.1 ± 0.5 and 1.4 ± 0.1 g/L, respectively. Neither DHA nor glycerol were detected in the fermentation broth. Thus, redirecting the native glycerol catabolic pathway failed for DHA or glycerol production.

As the DHA phosphate hydrolysis reaction has a high negative ΔrG (No. 4 in Table 1). *hdpA* of *C. glutamicum* was heterologously expressed in *Kp* Δ*tpiA* to construct *Kp* Δ*tpiA*-*hdpA*. This strain was cultured in flasks and results are shown in Fig. 2.

Cell growth of *Kp* Δ*tpiA*-*hdpA* was slower than that of *Kp* Δ*tpiA*. The highest cell density was only 0.5 ± 0.04 OD units. Glucose was exhausted and 2.0 ± 0.1 g/L of 2,3-butanediol and 1.5 ± 0.1 g/L of acetoin were produced after 21 h of cultivation. DHA was produced in the process, and its concentration was continuously increasing, and a final tier of 7.0 ± 0.3 g/L was obtained. 2.5 g/L of glycerol was also generated in the process. It should be pointed out that glycerol synthesis started later than DHA synthesis. Glycerol synthesis commenced after 15 h of cultivation, at this time the DHA concentration reached 6.7 g/L. *Kp hdpA*, wild type strain with overexpression of *hdpA*, was also cultured. But cell growth and metabolite production of this strain were similar to those of the wild type strain. No DHA nor glycerol was produced by *Kp hdpA* (data not shown). In summary, a redirected glycerol catabolism pathway was set up based on the knock-out of *tpiA* and overexpression of *hdpA*, and DHA and glycerol were produced from glucose through this pathway.

### Blocking by-products synthesis in DHA production

**Fig. 3:**
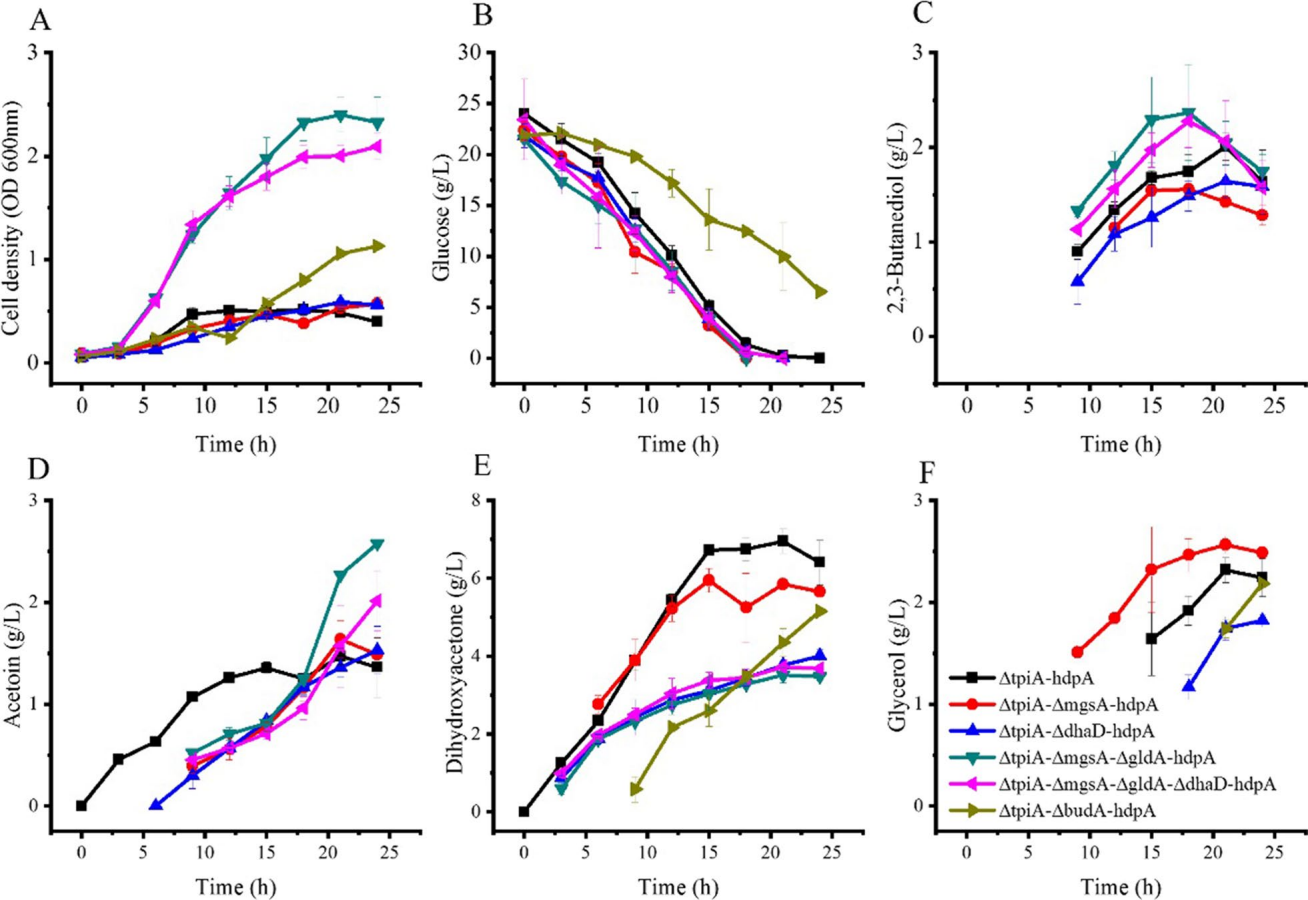
Growth and product formation of *K. pneumoniae* strains grown in shake flasks for reduce by-products of DHA production. ΔtpiA*-*hdpA: *Kp* Δ*tpiA-hdpA*; ΔtpiA-ΔmgsA-hdpA: *Kp* Δ*tpiA-*Δ*mgsA-hdpA*; ΔtpiA-ΔdhaD-hdpA: *Kp* Δ*tpiA-*Δ*dhaD-hdpA*; ΔtpiA-ΔmgsA-ΔgldA-hdpA: *Kp* Δ*tpiA-*Δ*mgsA-*Δ*gldA-hdpA*; ΔtpiA-ΔmgsA-ΔgldA-ΔdhaD-hdpA: *Kp* Δ*tpiA-*Δ*mgsA-*Δ*gldA-*Δ*dhaD-hdpA*; ΔtpiA-ΔbudA-hdpA: *Kp* Δ*tpiA-*Δ*budA-hdpA*; Data points are the average of n = 3; error bars represent standard error about the mean

Metabolites of *Kp* Δ*tpiA*-*hdpA* include DHA, glycerol, 2,3-butanediol and acetoin. If we consider DHA as the target product, then other metabolites are all by-products. *dhaD* and *gldA* encoding isoenzymes of glycerol dehydrogenase that catalyse the conversion between DHA and glycerol. *budA* encoding acetolactate synthase, which is a key enzyme of 2,3-butanediol and acetoin synthesis pathway. Besides, DHA can be converted to methylglyoxal and further be converted to pyruvate (Fig. 1). Methylglyoxal synthase is encoded by *mgsA*. These key genes were knocked out individually or combined and pDK6-hdpA was transformed to obtain *Kp* Δ*tpiA*-Δ*mgsA-hdpA*, *Kp* Δ*tpiA*-Δ*dhaD-hdpA*, *Kp* Δ*tpiA*-Δ*mgsA*-Δ*gldA-hdpA*, *Kp* Δ*tpiA*-Δ*mgsA*-Δ*gldA*-Δ*dhaD-hdpA*, and *Kp* Δ*tpiA*-Δ*budA-hdpA*. These strains and the *Kp* Δ*tpiA-hdpA* were cultured in flasks and results are shown in Fig. 3.

Cell growth, glucose consumption, 2,3-butanediol and acetoin production of *Kp* Δ*tpiA-*Δ*mgsA-hdpA* were similar to those of *Kp* Δ*tpiA-hdpA.* 5.6 ± 0.1 g/L of DHA and 2.5 ± 0.1 g/L of glycerol were produced by this strain. While, 6.4 ± 0.5 g/L of DHA and 2.2 ± 0.2 g/L of glycerol were produced by *Kp* Δ*tpiA-hdpA.* These results indicated that blocking the pathway of DHA to methylglyoxal had no positive effect on DHA production.

Glycerol production by *Kp* Δ*tpiA-*Δ*dhaD-hdpA* was reduced, with the titer of 1.8 ± 0.1 g/L. Whereas, no glycerol was produced by *Kp* Δ*tpiA-*Δ*mgsA-*Δ*gldA-hdpA*. *Kp* Δ*tpiA-*Δ*mgsA-*Δ*gldA-*Δ*dhaD-hdpA* also produced no glycerol. However, DHA production by these three strains were all reduced remarkably, rather than increased. Cell growths of *Kp* Δ*tpiA-*Δ*mgsA-*Δ*gldA-hdpA* and *Kp* Δ*tpiA-*Δ*mgsA-*Δ*gldA-*Δ*dhaD-hdpA* were enhanced, with final cell densities of 2.3 ± 0.2 and 2.1 ± 0.1 OD units, which were about four times that of *Kp* Δ*tpiA-hdpA*.

Neither 2,3-butanediol nor acetoin were synthesized by *Kp* Δ*tpiA-*Δ*budA-hdpA*. Glucose consumption and cell growth of this strain became slow, and 6.5 g/L of glucose was still unused in the broth after 24 h of cultivation. Glycerol produced by this strain was similar to that of *Kp* Δ*tpiA-hdpA*. However, DHA titer of this strain was lower than that of strain *Kp* Δ*tpiA-hdpA*. Thus, blocking the 2,3-butanediol synthesis pathway had no positive effect on DHA and glycerol production.

These efforts to reduce glycerol or other by-products production all failed to enhance the level of DHA.

### Blocking the reaction of glyceraldehyde 3-phosphate to 1,3-bisphospho-glycerate for DHA production

**Fig. 4:**
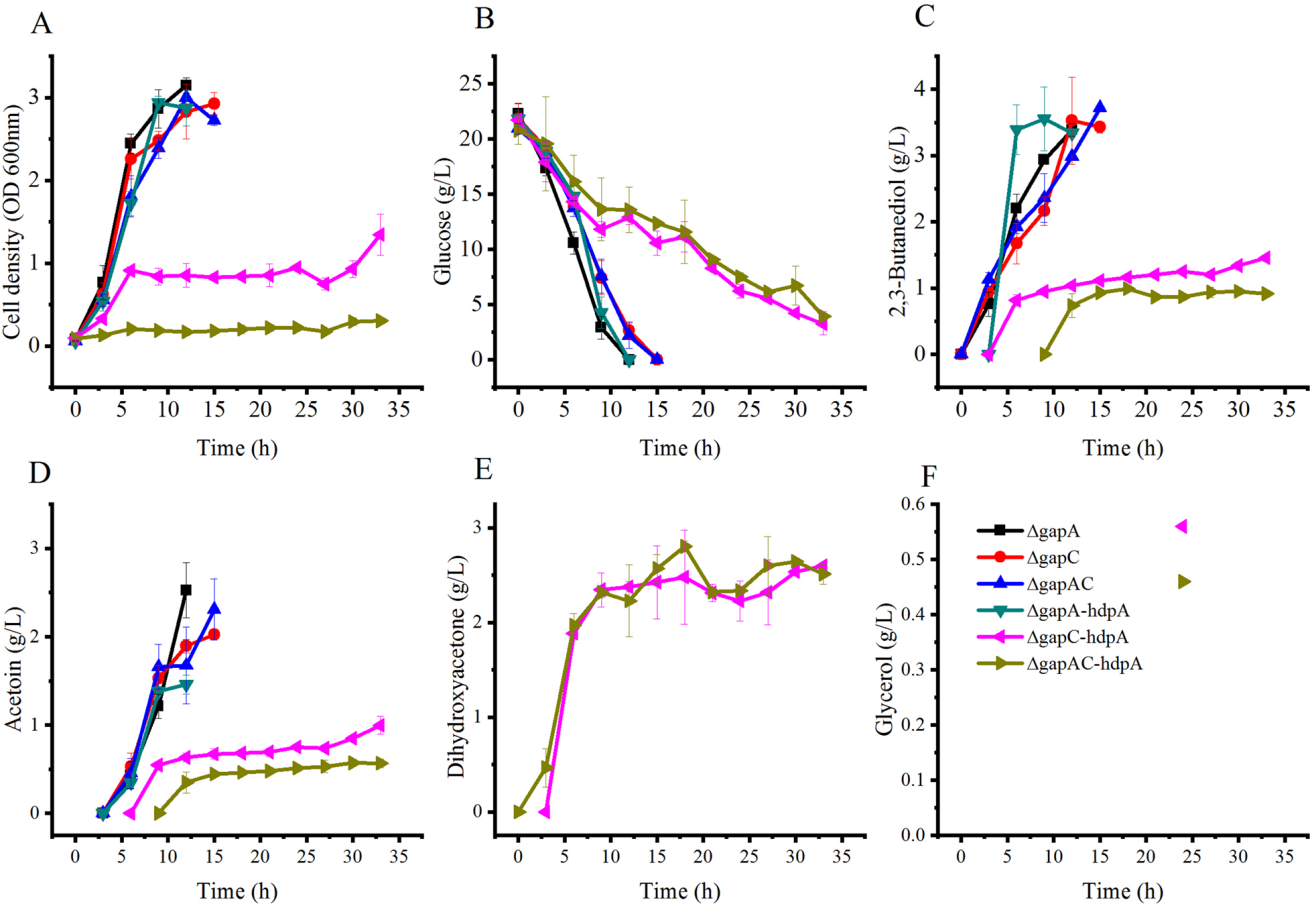
DHA production by glyceraldehyde-3-phosphate dehydrogenase disruption strains of *K. pneumoniae* grown in shake flasks. ΔgapA: *Kp* Δ*gapA*; ΔgapC: *Kp* Δ*gapC*; ΔgapAC: *Kp* Δ*gapC-*Δ*gapA*; ΔgapA *-*hdpA*: Kp* Δ*gapA-hdpA*; ΔgapC*-*hdpA: *Kp* Δ*gapC-hdpA*; ΔgapAC*-*hdpA*:Kp* Δ*gapC-*Δ*gapA-hdpA*. Data points are the average of n = 3; error bars represent standard error about the mean

The knock-out of *tpiA* prevented further catabolism of DHA phosphate in the glycolysis and resulted in its conversion to DHA. Glyceraldehyde-3-phosphate dehydrogenase catalyses the conversion of glyceraldehyde 3-phosphate to 1,3-bisphospho-glycerate. It was suspected, that blocking further catabolism of glyceraldehyde 3-phosphate might also result in DHA phosphate flow to DHA. Glyceraldehyde-3-phosphate dehydrogenase has three isoenzymes as noted in the genome of *Klebsiella variicola* 342, and they are encoded by *gap*, *gapA* and *gapC*, respectively [19]. The genome of this strain was highly homologous to that of *K.pneumoniae* CGMCC 1.6366 used in this study. However, only *gapA* and *gapC* were found in the genome of *K.pneumoniae* CGMCC 1.6366. The two genes were knocked out individually and combined to get *Kp* Δ*gapA, Kp* Δ*gapC* and *Kp* Δ*gapA-*Δ*gapC*. pDK6-*hdpA* was transformed into these genes knock-out strains to get the corresponding strains. These strains were cultured in flasks and results were shown in Fig. 4.

Physiological characteristics of *Kp* Δ*gapA* were comparable to that of wild-type strain (data shown in Fig. 2). 23 g/L of glucose was exhausted by these strains after 12 h of cultivation. 2,3-Butanediol and acetoin were the main products of this strain, with the titer of 3.3 ± 0.1 and 2.5 ± 0.3 g/L, respectively. Overexpression of *hdpA* in *Kp* Δ*gapA* failed for DHA or glycerol production. 3.3 ± 0.1 g/L of 2,3-butanediol and 1.5 ± 0.1 g/L of acetoin were produced by *Kp* Δ*gapA-hdpA*, which were similar to those of wild-type strain.

Cell growth of *Kp* Δ*gapC* was slightly slower compared to that of the wild-type strain, and glucose was exhausted after 15 h of cultivation. 2,3-Butanediol and acetoin produced by this strain were similar to those of wild-type stain. Physiological characteristics of *Kp* Δ*gapA-*Δ*gapC* were nearly the same as those of *Kp* Δ*gapC.*

Over-expression of *hdpA* in *Kp* Δ*gapC* has a distinct effect on the host cell. Cell growth of *Kp* Δ*gapC-hdpA* was very slow. After 33 h of cultivation, 3 g/L of glucose was still unused in the broth, and the cell density was lower than 1 OD unit during most periods of the process.

2,3-Butanediol and acetoin produced by *Kp* Δ*gapC-hdpA* were reduced to 1.4 ± 0.1 and 1.0 ± 0.1 g/L, respectively. These metabolites were mainly synthesized in the beginning 6 h of cultivation. 2.3 ± 0.2 g/L of DHA was produced after 9 h of cultivation, and after that its concentration had no distinct change. Glycerol was produced in the process, but its level was very low. 0.56 g/L of glycerol was detected after 24 h of cultivation. Metabolites of *Kp* Δ*gapA-*Δ*gapC-hdpA* were very likely that of *Kp* Δ*gapC-hdpA*. But the growth of this strain was very weak, and the highest cell density was only 0.2 OD unit.

Blocking the conversion of glyceraldehyde 3-phosphate to 1,3-bisphospho-glycerate and over-expression of the *hdpA* lead to DHA and glycerol production. However, the levels of DHA and glycerol produced by *Kp* Δ*gapC-hdpA* or *Kp* Δ*gapA-*Δ*gapC-hdpA* were lower than those produced by *Kp* Δ*tpiA-hdpA*. This indicated that blocking further catabolism of DHA phosphate in the cell by knocking out of *tpiA* or *gapC* were both effective for DHA and glycerol production through the redirected glycerol catabolism pathway.

### The effect of disruption of DHA kinases on DHA production

**Fig. 5:**
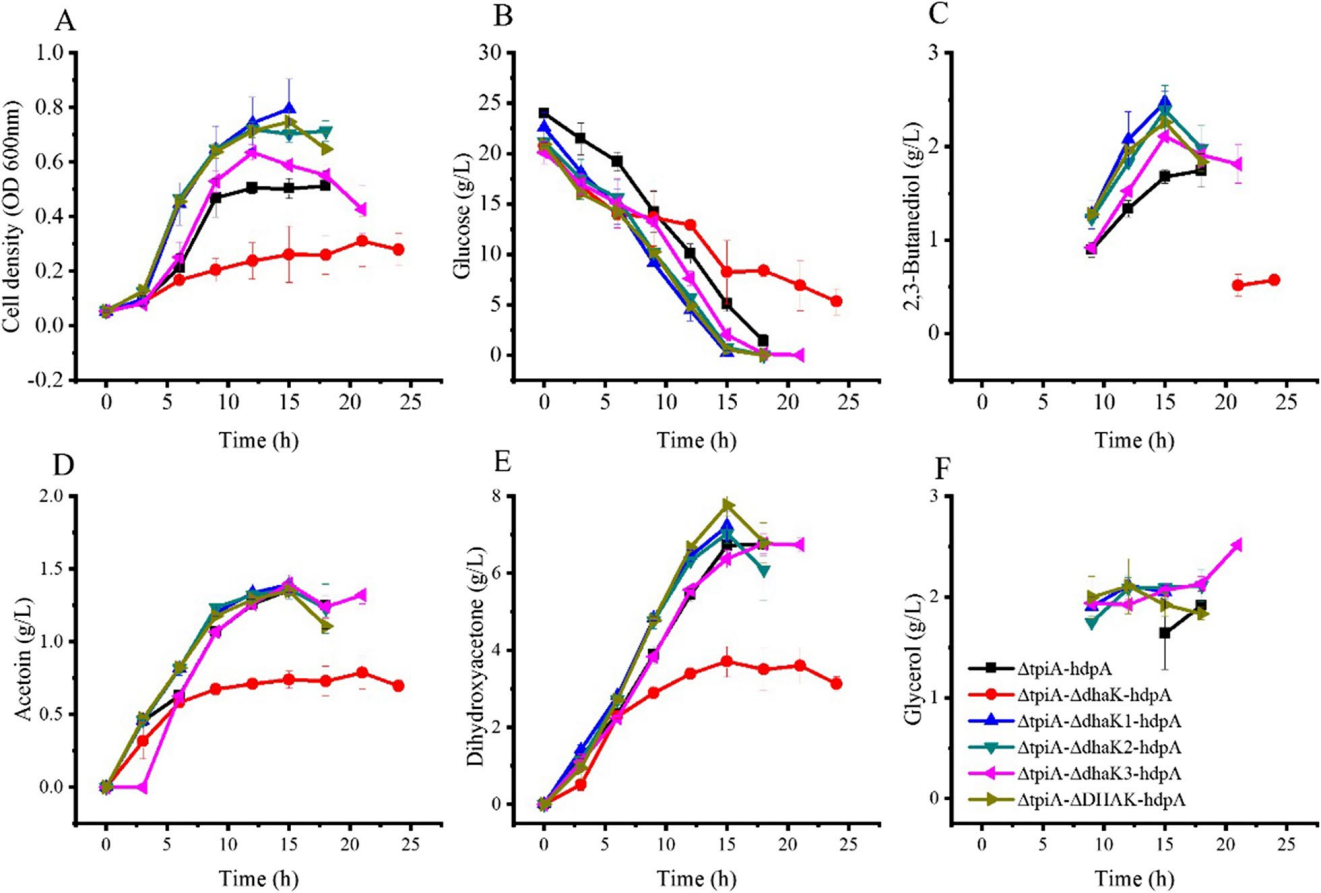
Growth and product formation of *K. pneumoniae* strains that knocked out genes that encoding isoenzymes of DHA kinases in shake flasks. ΔtpiA*-*hdpA: *Kp* Δ*tpiA-hdpA*; ΔtpiA*-*ΔdhaK-hdpA: *Kp* Δ*tpiA-*Δ*dhaK-hdpA*; ΔtpiA*-*ΔdhaK1-hdpA: *Kp* Δ*tpiA-*Δ*dhaK1-hdpA*; ΔtpiA*-*ΔdhaK2-hdpA: *Kp* Δ*tpiA-*Δ*dhaK2-hdpA*; ΔtpiA*-*ΔdhaK3-hdpA: *Kp* Δ*tpiA-*Δ*dhaK3-hdpA*; ΔtpiA*-*ΔDHAK-hdpA: *Kp* Δ*tpiA-*Δ*DHAK-hdpA*. Data points are the average of n = 3; error bars represent standard error about the mean

DHA kinases and DHA phosphate dephosphorylase catalyse the reaction of DHA phosphorylated and DHA phosphate dephosphorylated, respectively. If they both working at the same time, they form an futile cycle in the cell. To erase this reaction cycle, the subunits of the two kinases were disrupted individually and *hdpA* was expressed to obtain the following strains: *Kp* Δ*tpiA-*Δ*dhaK-hdpA*, *Kp* Δ*tpiA-*Δ*dhaK1-hdpA*, *Kp* Δ*tpiA-*Δ*dhaK2-hdpA*, *Kp* Δ*tpiA-*Δ*dhaK3-hdpA*, and *Kp* Δ*tpiA-*Δ*DHAK-hdpA*. *Kp* Δ*tpiA-*Δ*DHAK-hdpA* was a strain where *dhaK*, *dhaK1*, *dhaK2* and *dhaK3* were all knocked out. These strains were cultured in flasks and results are shown in Fig. 5.

Cell growth of *Kp* Δ*tpiA-*Δ*dhaK-hdpA* was slow compared with that of *Kp* Δ*tpiA-hdpA*. The final cell density of this strain was 0.3 ± 0.1 OD units. Accordingly, glucose consumption and metabolites productivity were all in low rates. 0.5 ± 0.1 g/L of 2,3-butanediol, 0.8 ± 0.1 g/L of acetoin and 3.6 ± 0.5 g/L of DHA were produced by this strain, while glycerol was not detected in the process.

Cell growth of *Kp* Δ*tpiA-*Δ*dhaK1-hdpA*, *Kp* Δ*tpiA-*Δ*dhaK2-hdpA* and *Kp* Δ*tpiA-*Δ*DHAK-hdpA* were faster than that of *Kp* Δ*tpiA-hdpA*. The highest cell densities of these strains were 0.8 ± 0.1, 0.6 ± 0.1 and 0.7 ± 0.1 OD units, respectively. Glucose consumption rate of these strains were faster than that of *Kp* Δ*tpiA-hdpA*. 2,3-Butanediol titers of these strains were 2.5 ± 0.1, 2.4 ± 0.2 and 2.3 ± 0.1 g/L, respectively. Acetoin titer of these strains were similar to that of *Kp* Δ*tpiA-hdpA*. DHA produced by these strains were 7.2 ± 0.4, 7.0 ± 0.2 and 7.8 ± 0.3 g/L, respectively. *Kp* Δ*tpiA-*Δ*DHAK-hdpA* had the highest DHA titer among these strains. Glycerol titers of these strains were similar to that of *Kp* Δ*tpiA-hdpA*, but productivities were higher than that of *Kp* Δ*tpiA-hdpA*. Cell growth and glucose consumption of *Kp* Δ*tpiA-*Δ*dhaK3-hdpA* were similar to that of *Kp* Δ*tpiA-hdpA*. Glycerol produced by this strain was higher than *Kp* Δ*tpiA-hdpA*.

The DHA and glycerol production by these strains are summarized in Table 2.

**Table 2:** DHA and glycerol production by strains of inactive isoenzymes of DHA kinase

Strains	Time (h)	Glucose consumed (g/L)	DHA (g/L)	Glycerol (g/L)	DHA conversion ratio (mol/ mol)	Glycerol conversion ratio (mol/ mol)	Total conversion ratio (mol/ mol)
ΔtpiA-hdpA	18	22.6	6.7	1.9	0.60	0.17	0.76
ΔtpiA-Δdhak-hdpA	24	15.4	3.1	0	0.41	0	0.41
ΔtpiA-Δdhak1-hdpA	15	22.4	7.2	2.0	0.65	0.18	0.83
ΔtpiA-Δdhak2-hdpA	15	20.4	7.0	2.1	0.69	0.20	0.89
ΔtpiA-Δdhak3-hdpA	18	20.0	6.8	2.1	0.68	0.21	0.88
ΔtpiA-ΔDHAK-hdpA	15	20.4	7.8	1.9	0.76	0.19	0.95

Except *Kp* Δ*tpiA*-Δ*dhak*-*hdpA*, the conversion ratio of glucose to DHA and glycerol in these strains were all improved compared with that of *Kp* ΔtpiA-hdpA. The total conversion ratio of glucose to DHA and glycerol was 0.95 in *Kp* Δ*tpiA-*Δ*DHAK-hdpA*, which was nearly the maximum theoretical conversion ratio. This strain also had the highest DHA and glycerol productivity and was selected for further investigation.

### DHA production by ***Kp*** Δ***tpiA-***Δ***DHAK-hdpA*** using different carbon sources

*Kp* Δ*tpiA-*Δ*DHAK-hdpA* was cultured in flasks with glucose, xylose, sucrose and fucose as the main carbon source, and results are shown in Additional file 1: Fig. S1. All these carbon sources can be used by the cell for DHA and glycerol production. The conversion ratios of glucose, sucrose and fucose to DHA and glycerol were similar. The conversion ratio of xylose to DHA and glycerol was lower than others, with the value of 0.38 mol/mol. These results were reasonable. Sucrose was hydrolysed to form glucose and fructose, and the two monosaccharoses were all catabolised through the glycolysis pathway. Fucose is a monosaccharose that is rich in marine algae. In *K. pneumoniae*, fucose was converted to fuculose and further to fuculose-phosphate. DHA-phosphate and lactaldehyde was formed from fuculose-phosphate with the catalysis of an aldolase. Xylose was catabolised through the Pentose phosphate pathway, only part of carbon was converted to DHA-phosphate and resulted a low conversion ratio to DHA and glycerol. While, more carbon was used for cell growth, and a high cell density was obtained with xylose as the carbon source. It can be concluded that any carbon source that can be catabolised to form DHA-phosphate is suitable for DHA and glycerol production by *Kp* Δ*tpiA-*Δ*DHAK-hdpA*.

### Fermentation parameters optimization

#### Culture pH optimization

**Fig. 6:**
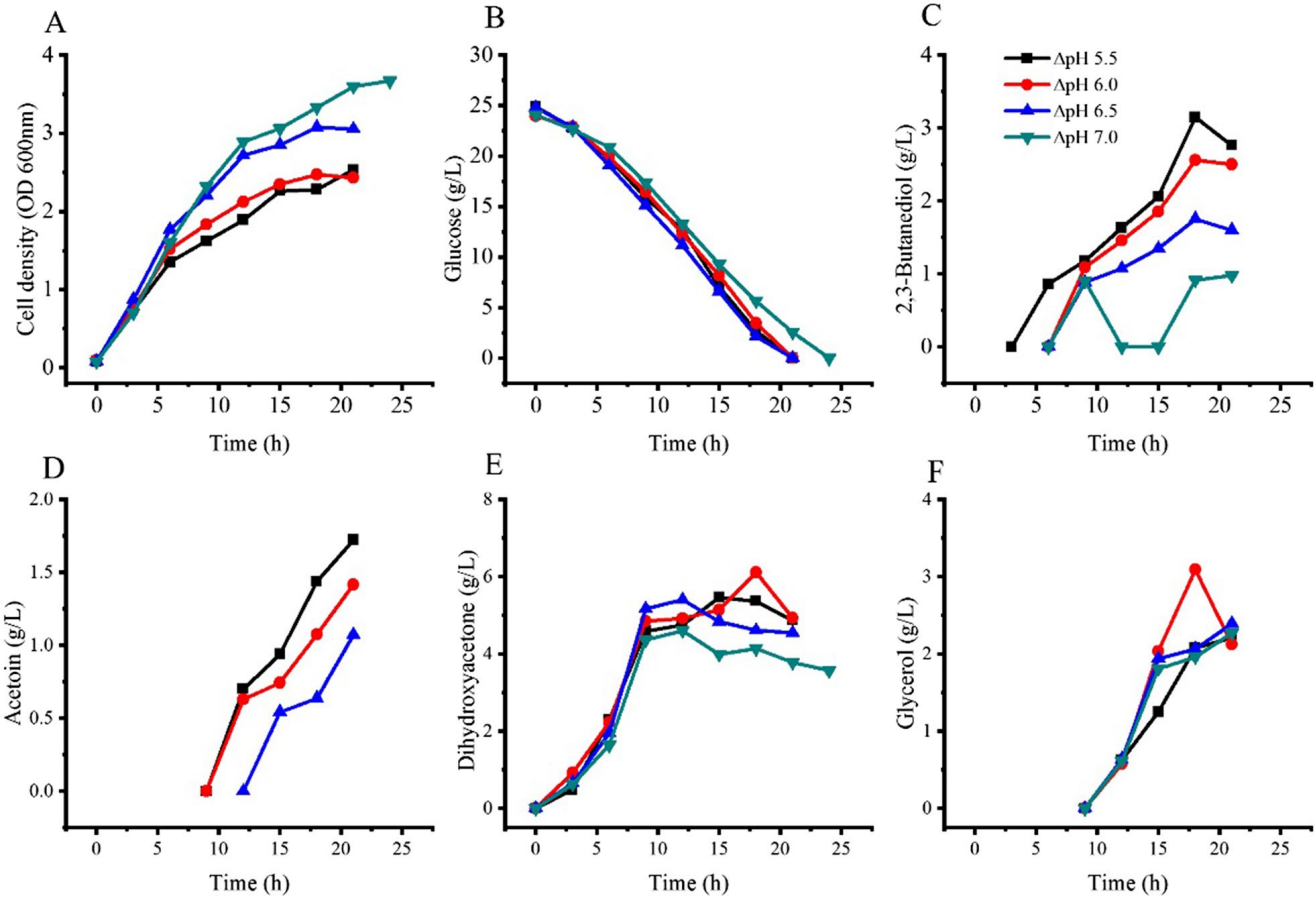
Growth and product formation of *Kp* Δ*tpiA-*Δ*DHAK-hdpA* in bioreactors at different culture pH. The strain was grown in stirred tank bioreactors operated at an agitation rate of 250 rpm and an aeration rate of 2 L/min

*Kp* Δ*tpiA-*Δ*DHAK-hdpA* was cultured in 5 L bioreactors with fermentation medium, where the culture pH was stabilized at 5.5, 6.0, 6.5, and 7.0. The air flow rate was set at 2 L/min and the stirring rate of the bioreactor was set at 250 rpm. Fermentation results are presented in Fig. 6.

Cell growth was positively related to the culture pH. The lowest and the highest cell densities were obtained at the culture pH 5.5 and 7.0, respectively. All cell densities obtained were higher than that in flask culture (shown in Fig. 5). Glucose consumption rates were similar in the range of pH 5.5–6.5. Glucose consumption rate in culture pH 7.0 was low compared with other conditions, and it took 24 h to use all the glucose supplied, which was slower than the consumption in flask culture.

2,3-Butanediol and acetoin production was inversely related to the culture pH. 2.7 g/L of 2,3-butanediol and 1.7 g/L of acetoin were produced in culture pH 5.5, while only 0.9 g/L of 2,3-butanediol was produced in culture pH 7.0, and no acetoin was detected at this condition.

DHA was produced from the beginning of cultivation, and quickly reached its high levels at 9 h of cultivation. Glycerol production started at 9 h, and its levels increased towards the end of the process. 6.1 g/L of DHA was produced at culture pH 6.0. Lower or higher culture pH all resulted in lower DHA titers. The lowest DHA titer was obtained at culture pH 7.0, reaching 4.1 g/L. Glycerol titers were in agreement with DHA titers. The highest glycerol titer was 3.1 g/L, which was obtained at culture pH 6.0.

DHA and glycerol titers and the substrate conversion ratios of these experiments are summarized in Table 3. The conversion ratio of glucose to DHA and glycerol both had the highest values at culture pH 6.0, and the total value was 0.90. Productivities of DHA and glycerol in culture pH 6.0 were the fastest among all experimental conditions. Thus pH 6.0 was selected as the optimized culture pH.

**Table 3:** DHA and glycerol production by *Kp* Δ*tpiA-*Δ*DHAK-hdpA* at different culture pH

Culture pH	Time (h)	Glucose consumed (g/L)	DHA (g/L)	Glycerol (g/L)	DHA conversion ratio (mol/ mol)	Glycerol conversion ratio (mol/ mol)	Sum conversion ratio (mol/ mol)
5.5	18	22.2	5.4	2.1	0.49	0.19	0.68
6.0	18	20.4	6.1	3.1	0.60	0.30	0.90
6.5	18	20.5	4.6	3.1	0.45	0.30	0.75
7.0	21	21.5	3.8	2.3	0.35	0.21	0.57

Oxygen supplementation.

**Fig. 7:**
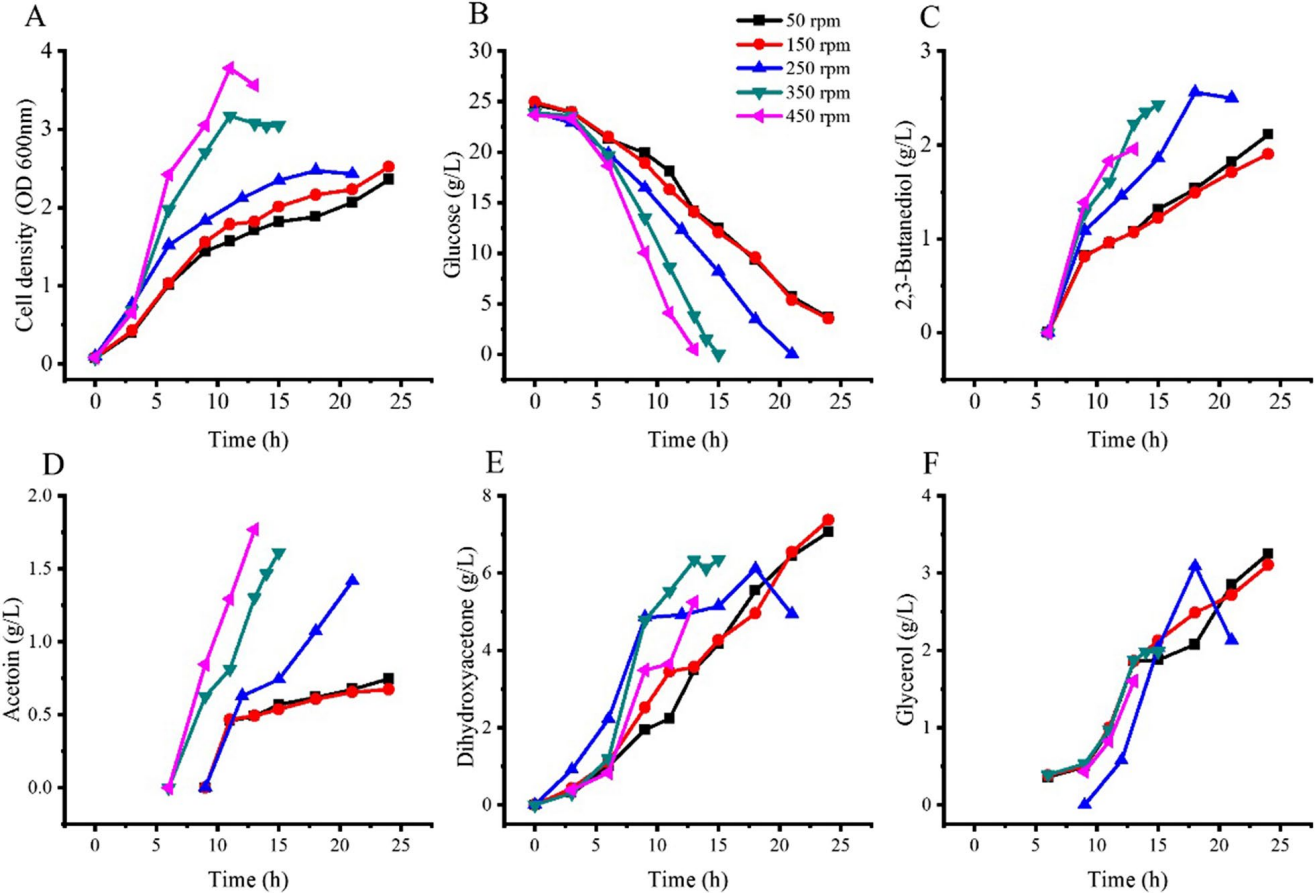
Growth and product formation of *Kp* Δ*tpiA-*Δ*DHAK-hdpA* in bioreactors with different stirring rates. The strain was grown in stirred tank bioreactors and the aeration rate set at 2 L/min, culture pH 6.0. (250 rpm data were the same shown in Fig. 6)

*Kp* Δ*tpiA-*Δ*DHAK-hdpA* was cultured in 5L bioreactors. The air flow rate was set at 2L/min and the stirring rate of the bioreactor was set at 50, 150, 250, 250 and 450 rpm to obtain different aerobic conditions. Culture pH were set at 6.0. Fermentation results are presented in Fig. 7.

Cells growth and glucose consumption had a positive relationship with stirring rates. The higher the stirring rate the higher the cell density and the glucose consumption rate. 3.6 OD unit was obtained at a stirring rate of 450 rpm. The lowest cell density was 2.3 OD unit at stirring rate of 50 rpm. Glucose was exhausted after 13 h of cultivation at a stirring rate of 450 rpm, followed by stirring rate of 350 and 250 rpm. Whilst 3.5 and 3.6 g/L of glucose were still unused at stirring rate of 50 and 150 rpm after 24 h of cultivation. 2,3-Butanediol and acetoin productivities were improved with the increase of the stirring rate. Stirring rate of 50 and 150 rpm had the lowest titers of 2,3-butanediol and acetoin.

The productivity of DHA at stirring rate of 50 and 150 rpm conditions was similar, and these were the lowest value among all experimental conditions. However, the final titers of DHA were the highest, with the value of 7.1 g/L and 7.4 g/L, respectively. At the same time, 3.3 g/L and 3.1 g/L of glycerol were produced, which were also the highest value among all agitation conditions.

The final titers of DHA and glycerol and substrate conversion ratios at different oxygen supplementation are summarized in Table 4. The total conversion ratio of glucose to DHA and glycerol was 0.97 and 0.98 at stirring rate of 50 and 150 rpm conditions. It means nearly all DHA phosphate were transferred to DHA and glycerol in these conditions. In contrast, the total conversion ratio of glucose to DHA and glycerol was 0.58 at stirring rate of 550 rpm. Thus, lower oxygen supplementation favored DHA and glycerol production.

**Table 4:** DHA and glycerol production by *Kp* Δ*tpiA-*Δ*DHAK-hdpA* at different stirring rates

Stirring rate	Time (h)	Glucose consumed (g/L)	DHA (g/L)	Glycerol (g/L)	DHA conversion ratio (mol/ mol)	Glycerol conversion ratio (mol/ mol)	Total conversion ratio (mol/ mol)
50	24	21.1	7.1	3.2	0.67	0.30	0.97
150	24	21.4	7.4	3.1	0.69	0.29	0.98
250	21	20.4	6.1	3.1	0.60	0.30	0.90
350	15	23.9	6.4	2.0	0.54	0.17	0.70
450	13	23.2	5.3	1.6	0.44	0.13	0.58

### Fed batch fermentation

**Fig. 8:**
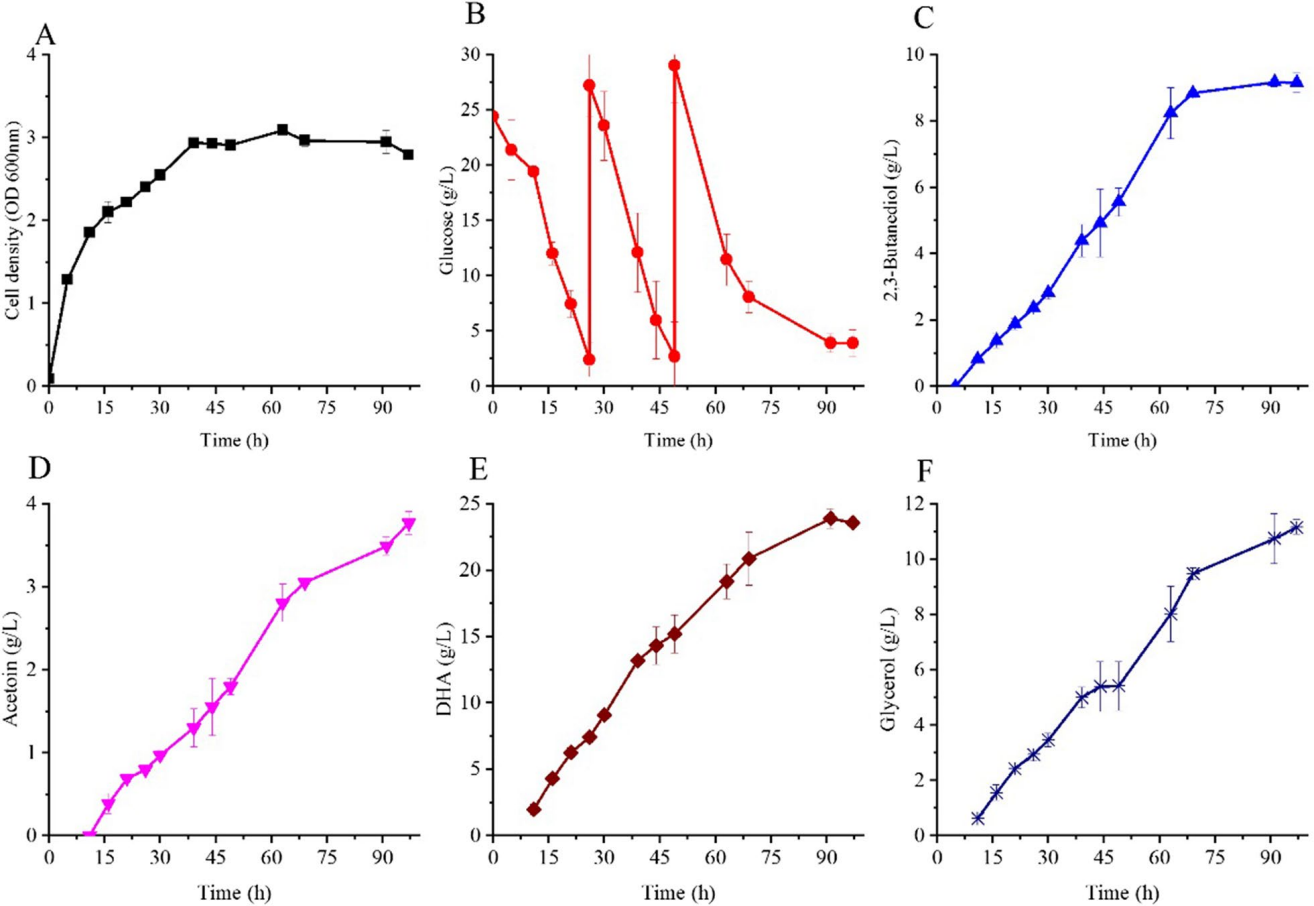
Growth and product formation of *Kp* Δ*tpiA-*Δ*DHAK-hdpA* in fed batch fermentation. Cells were grown in a stirred tank bioreactor operated at aeration rate of 2 L/min. In the first 6 h the culture pH and stirring rate were set at 7.0 and 450 rpm. After 6 h the culture pH and stirring rate were set at 6.0 and 150 rpm. Glucose was fed at 26 and 49 h of cultivation. Data points are the average of n = 3; error bars represent standard error about the mean

High culture pH and high oxygen supplementation favor for cell growth, but the conversion ratio of glucose to DHA and glycerol were low. Whereas low culture pH and low oxygen supplementation favor a high substrate conversion ratio, with low cell growth rate. To solve this inverse relationship, a two- phase culture strategy was used. In the beginning 6 h of culture, the culture pH and stirring rate were set at 7.0 and 450 rpm, which were favorable for cell growth. Then, the culture pH and stirring rate were switched to 6.0 and 150 rpm, since this condition favored DHA and glycerol production. Fed batch fermentations were conducted to obtain a superior final product level. Fermentation results are presented in Fig. 8.

In fed batch fermentation, cells were rapidly growing during the first 16 h of cultivation and the cell density reached to 2.1 OD units. After about 30 h cells entered a stationary phase and the highest cell density was reached at 63 h of culture (3.1 OD units). The glucose concentration in the broth dropped to 2.4 g/L after 26 h. After addition of highly concentrated glucose solution due to this initial drop, the glucose concentration increased to 27.2 g/L. At 49 h, another glucose feed had to be performed. DHA was produced in the broth and its concentration continued to increase. The highest DHA titer of 23.9 g/L was achieved after 91 h of cultivation. Glycerol production followed production of DHA and its titer was 10.8 g/L after 91 h of cultivation. The main by-products of this process were 2,3-butanediol and acetoin, reaching a titer of 9.2 and 3.5 g/L, respectively. Based on the production of metabolites and biomass, the carbon balance calculated for DHA, glycerol, 2,3-butanediol, acetoin and biomass were 33%, 15%, 17%, 7%, and 2%, respectively. The carbon recovery ratio was 74%. The missing carbon was likely to be CO_2_ generated in the process and additionally a small amount of other chemicals. The total conversion ratio of DHA and glycerol from glucose was 0.97 mol/mol.

DHA is a toxic chemical to the cell and high levels of DHA are known to inhibit the activity of cells [20]. The inhibitory effect of DHA on the wild-type *K. pneumoniae* was determined and results are shown in Additional file 1: Fig. S2. As expected cell growth was inhibited by DHA. With the concentration of DHA in the broth increasing, the inhibitory effect became more obvious. Cell growth was totally stopped at 22 /L of DHA. The 23.9 g/L of DHA obtained in fed batch fermentation exceeded the highest level of DHA that cell can tolerate. Thus, cells might have lost metabolic activities in this condition; preventing the DHA titer to increase further.

## Discussion

### Redirection of glycerol catabolic pathway for DHA and glycerol production

In our previous research about the isoenzymes of DHA kinase, it was found that disruption of DHA kinase I had no effect on glycerol catabolism. Whereas the activity of DHA kinase II was essential for glycerol catabolism [8]. This agrees with the thermodynamic analysis of the glycerol catabolic pathway shown in Table 1. DHA kinase II should be the functional enzyme in glycerol catabolism as the reaction of glycerol conversion to DHA that is catalysed by DhaD prefers the opposite direction. The glycerol catabolism pathway was favoured by the high negative ΔrG of the reaction of DHA phosphorylation. Thus, DHA concentration in the cell was at a very low level and could not accumulate in the broth. An *Saccharomyces cerevisiae* strain was constructed for DHA production, and a NAD-dependent glycerol dehydrogenase was used for DHA formation from glycerol. This strain produced very low level of DHA with the titer of 0.7 g/L [21]. The reaction of glycerol to DHA combined with NADH formation has the same ΔrG, regardless of different glycerol dehydrogenase used. Thus, it was not possible to achieve high level of DHA production using NADH-linked glycerol dehydrogenase. However, DHA could be produced from glycerol by the cells of *G. oxydans* [12]. The enzyme catalysis this reaction was a membrane-bound enzyme and located in the periplasmic space. This glycerol dehydrogenase uses PQQ instead of NAD as the cofactor. The ΔrG of this reaction was negative, and the reaction equilibrium favors DHA formation.

Glucose is a commonly used carbon source for bacterial growth. DHA phosphate is an intermediate of the glycolysis. Neither the wild-type *K. pneumoniae* nor the *Kp* Δ*tpiA* produce DHA or glycerol (Fig. 2) indicated that the native glycerol catabolism pathway was difficult to work in the opposite direction. *hdpA* encode a haloacid dehalogenase superfamily member that catalyzes dephosphorylation of DHA phosphate to produce DHA in *C. glutamicum* [22]. After *hdpA* was introduced to the cell, a considerable level of DHA and glycerol was produced by *Kp* Δ*tpiA*-*hdpA* (Fig. 2). This was reasonable, because the ΔrG of this redirected glycerol catabolism pathway was estimated to be − 37.3 kJ/mol (Table 1). Besides overexpression of *hdpA*, the disruption of *tpiA* was also essential for DHA and glycerol production. In the research of 1,3-propanediol production from glucose and use *E. coli* as the host cell the inactivation of *tpiA* lead to assembling the carbon pathway from DHA phosphate to 1,3-propanediol [23]. In that research DHA phosphate was first reduced to glycerol phosphate and the latter was converted to glycerol, glycerol-3-phosphate dehydrogenase and glycerol-3-phosphatase cloned from *Saccharomyces cerevisiae* were used to catalyse the reaction. Glycerol was synthesized in a similar way when *K. pneumoniae* was used as the host [24].

### GldA, rather than DhaD is the main enzyme that catalyses glycerol formation from DHA

GldA and DhaD are isoenzymes of glycerol dehydrogenase. The amino acid sequence of the two enzymes exhibits high identities, but their expression was different. *dhaD* is a gene in the *dha* operon. It was reported that the expression of the *dha* operon was induced by DHA [9], and DhaR and DhaK123 were involved in the induction process [8]. *gldA* is an independent gene in the genome of *K. pneumoniae*, and there are no regulatory genes nearby. It has been noted that the expression of *dhaD* and *gldA* were both at low levels when cells were cultured on glucose as carbon source [25]. The expression of *dhaD* was induced by glycerol but the expression of *gldA* was not subject to glycerol induction [25]. DHA and glycerol were produced by *Kp* Δ*tpiA-hdpA* with glucose as the carbon source. The results shown in Fig. 3 indicated GldA was the main functional enzyme catalysing the formation of glycerol, and the expression of *dhaD* was inhibited by glucose, even glycerol and DHA were present in the broth.

Glycerol was also produced in a report on DHA production from glucose by engineering *E. coli*. In that work the glycerol titer was reduced and DHA titer was increased by knocking out *gldA* [16]. This was different to the results obtained in this study. Glycerol production was reduced by the strains *Kp* Δ*tpiA-*Δ*mgsA-*Δ*gldA-hdpA* and *Kp* Δ*tpiA-*Δ*dhaD-hdpA*. However, DHA production was also reduced by these strains.

### DHA production by blocking the conversion of glyceraldehyde-3-phosphate to 1,3-bisphospho-glycerate

*gapA* and *gapC* encode isoenzymes of glyceraldehyde-3-phosphate dehydrogenase. Physiological characteristics of *Kp* Δ*gapA*, *Kp* Δ*gapC* and *Kp* Δ*gapC-*Δ*gapA* were like that of the wild-type strain. This indicated, other isoenzymes or pathways might exist in the cell that are responsible for glyceraldehyde-3-phosphate catabolism. D-erythrose-4-phosphate dehydrogenase, an enzyme involved in vitamin B6 metabolism and encoded by *gapB*, exhibit glyceraldehyde-3-phosphate dehydrogenase in *E. coli* [26]. Similarly, an *epd* encoded D-erythrose-4-phosphate dehydrogenase was noted in the genome of *K. pneumoniae* CGMCC 1.6366. Thus D-erythrose-4-phosphate dehydrogenase might be the likely candidate. DHA and glycerol were produced by *Kp* Δ*gapC-hdpA* and *Kp* Δ*gapC-*Δ*gapA-hdpA*, but not by *Kp* Δ*gapA-hdpA*. This indicated, the isoenzyme of glyceraldehyde-3-phosphate dehydrogenase that is encoded by *gapC* was the principal functional enzyme in the cell. Inactivation of GapC blocked or partly blocked the further metabolism of glyceraldehyde-3-phosphate. The strategy of down-regulation of glyceraldehyde-3-phosphate dehydrogenase was done to assemble the carbon pathway from DHA phosphate to 1,3-propanediol, and this strategy was better than the knockout of *tpiA* in terms of 1,3-propanediol yield [23]. It was different to the DHA and glycerol production in this study, where the knockout of *tpiA* resulted in a higher yield of DHA and glycerol.

### Roles of DHA kinases on DHA production

DHA kinase I and DHA kinase II are isoenzymes, which catalyse the formation of DHA phosphate from DHA. Beside the catalysis function, subunits of DHA kinase II are involved in the regulation of the *dha* operon [8]. The DHA produced by *Kp* Δ*tpiA-*Δ*dhaK1-hdpA, Kp* Δ*tpiA-*Δ*dhaK2-hdpA, Kp* Δ*tpiA-*Δ*dhaK3-hdpA* and *Kp* Δ*tpiA-*Δ*DHAK-hdpA* were increased compared with that of *Kp* Δ*tpiA-hdpA* indicating that DHA kinase II was active in *Kp* Δ*tpiA-hdpA.* Whereas knocking out *dhaK* had no effect on glycerol catabolism [8] and cell growth and DHA production by *Kp* Δ*tpiA-*Δ*dhaK-hdpA* was decreased compared with *Kp* Δ*tpiA-hdpA*. The function of DHA kinase I in DHA and glycerol production from glucose remains unclear.

### DHA production by ***Kp*** Δ***tpiA-***Δ***DHAK-hdpA***

In the flasks culture of *Kp* Δ*tpiA-*Δ*DHAK-hdpA*, a total substrate conversion ratio of 0.95 was obtained. This result indicated nearly all the glucose was catabolised through the glycolysis pathway, and the DHA phosphate formed in the glycolysis was converted to DHA. Due to the lack of pH control in the flask the pH continuously decreased during the cultivation process. It was confirmed in the stirred tank bioreactors experiments that a low pH is favorable for DHA and glycerol production. pH 6.0 had the highest DHA and glycerol titers, but DHA and glycerol production at pH 5.5 were low. The likely reason was some gluconic acid and 2-ketogluconic acid were formed through the glucose oxidization pathway that is located in the periplasmic space, and acidic condition favor gluconic acid and 2-ketogluconic acid accumulation [1, 2]. At neutral pH conditions or high oxygen supplement conditions, cell growth of *Kp* Δ*tpiA-*Δ*DHAK-hdpA* were enhanced, but all metabolites were decreased. Instead of DHA phosphate, 3-phosphoglyceraldehyde and pyruvate were formed as intermediate of the glucose oxidization pathway [1]. Glucose catabolism through oxidization pathway rather than glycolysis will reduce the formation of DHA phosphate, and leads to low conversion ratio of glucose to DHA and glycerol.

In this study, 7.8 g/L of DHA and 1.9 g/L of glycerol were produced by *Kp* Δ*tpiA-*Δ*DHAK-hdpA* after 15 h of cultivation in flasks. The final titer, productivity and substrate conversion ratio obtained in this study were all higher than that in the report of DHA production by engineering *E. coli*. In that work 6.6 g/l of DHA was produced after 40 h of cultivation, and the substrate conversion ratio was 0.87 [16]. 23.9 g/L of DHA and 10.8 g/L of glycerol obtained in fed batch fermentation provides the basis for a novel and highly efficient way of DHA and glycerol production from glucose.

## Methods

### Strains, plasmids, and primers

Bacterial strains and plasmids used in this study are listed in Table 5. Primers used for PCR are listed in Additional file 1: Table S1.

**Table 5:** Strains and plasmids

Strains or plasmids	Relevant genotype and description	References or source
*Corynebacterium glutamicum* ATCC 13032		Lab stock
*E. coli* DH5α	Host of plasmid	Lab stock
*K. pneumoniae* CGMCC 1.6366	TUAC01 Wild type, Amp^r^	[7]
ΔtpiA	ΔtpiA, Apr^r^	This work
ΔtpiA-ΔmgsA	ΔmgsA, ΔtpiA, Apr^r^, Str^r^	This work
ΔtpiA-ΔmgsA-ΔgldA	ΔmgsA, ΔtpiA, ΔgldA, Apr^r^	This work
ΔtpiA-ΔmgsA-ΔgldA-ΔdhaD	ΔmgsA, ΔtpiA, ΔgldA, ΔdhaD, Apr^r^, Str^r^	This work
ΔtpiA-ΔdhaD	ΔdhaD,ΔtpiA, Apr^r^	This work
ΔtpiA-ΔbudA	ΔbudA, ΔtpiA, Apr^r^, Str^r^	This work
ΔgapA	ΔgapA, Apr^r^	This work
ΔgapC	ΔgapC, Apr^r^	This work
ΔgapA-ΔgapC	ΔgapA, ΔgapC, Apr^r^, Str^r^	This work
ΔtpiA-ΔdhaK	Δdhak, ΔtpiA, Apr^r^	This work
ΔtpiA-ΔdhaK1	Δdhak1, ΔtpiA, Apr^r^	This work
ΔtpiA-ΔdhaK2	Δdhak2, ΔtpiA, Apr^r^	This work
ΔtpiA-ΔdhaK3	Δdhak3, ΔtpiA, Apr^r^	This work
ΔtpiA-ΔDHAK	Δdhak, Δdhak123, ΔtpiA, Apr^r^	This work
Plasmids		
pIJ773	Apr^r^, *aac(3)IV* with FRT sites, 4334 bp	[27]
pIJ778	Str^r^, *aadA* with FRT sites, 4337 bp	[27]
pDK6	Kan^r^, lacI^Q^, tac, 5118 bp	[28]
pDK6-hdpA	Kan^r^, carries *hdpA*, 5.9kp	This work
pDK6-red	Kan^r^, carries λ-Red genes (gam, bet, exo) 7.1 kp	[29]
pDK6-flp	Kan^r^, carries the yeast FLP recombinase gene 6.3 kp	[29]
pMD18-T-simple	Amp^r^, TA cloning vector, 2692 bp	Takara
pMD18-T-ΔtpiA	Amp^r^, Apr^r^, carries part of *tpiA*	This work
pMD18-T-ΔmgsA	Amp^r^, Str^r^, carries part of *mgsA*	This work
pMD18-T-ΔgldA	Amp^r^, Apr^r^, carries part of *gldA*	This work
pMD18-T-ΔdhaD	Amp^r^, Str^r^, carries part of *dhaD*	This work
pMD18-T-ΔgapA	Amp^r^, Apr^r^, carries part of *gapA*	This work
pMD18-T-ΔgapC	Amp^r^, Apr^r^, carries part of *gapC*	This work

### Gibbs free energy reaction estimation

Gibbs free energy of metabolic reactions were estimated with the help of the eQuilibrator (http://equilibrator.weizmann.ac.il). eQuilibrator is a simple web interface designed to enable easy thermodynamic analysis of biochemical systems. All the substrate and cofactor were set at standard conditions with the concentration of 1 mol/L, pH 7.0 and ionic strength 0.1 mol/L.

### Knocking out genes in the genome of *K. pneumoniae*

For genes knock out, *K. pneumoniae* and *E. coli* were grown in Luria–Bertani (LB) medium at 37 °C. The antibiotics used in the selective medium were ampicillin (50 μg/mL), kanamycin (50 μg/mL), apramycin (50 μg/mL), and streptomycin (25 μg/mL).

#### *Kp* Δ*tpiA* construction

*Kp* Δ*tpiA* and other gene knock out strains constructions were generated using the Red recombinase associated gene replacement method as described previously with some modification [29]. The up and drown flanking sequences of *tpiA* gene in the genome of *K. pneumoniae* were amplified by PCR using the primer pair tpiA-up-s/a and tpiA-down-s/a. Apramycin resistance gene *aac(3)IV* was amplified with plasmid pIJ773 as the template using the primer pair tpiA-FRT-s/a. The up and drown flanking sequences of *tpi*A and *aac(3)IV* were ligated together with a ClonExpress Ultra One Step Cloning Kit® to generate a linear DNA containing the apramycin resistance gene *aac(3)IV* with 600 bp of *tpiA* homologous regions on both sides. The linear DNA was transformed into *K. pneumoniae* CGMCC 1.6366, which already hosted the plasmid pDK6-red. Homologous recombination between the linear DNA and the chromosome was facilitated by Red recombinase and led to *tpiA* deletion in the genome of *K. pneumoniae* to obtain *Kp* Δ*tpiA*.

#### *K. pneumoniae* ΔtpiA-Δdhak construction

*Kp* Δ*tpiA-*Δ*dhak* was constructed following the same way of *Kp* Δ*tpiA* construction, with *Kp* Δ*dhak* [8] replacing wild-type *K. pneumoniae* as the target strain.

The ORF of *hdpA* in *C. glutamicum* was amplified using the primer pair *hdpA*-s and *hdpA*-s. The PCR product was digested with restriction enzyme *EcoR I* and *BamH I*. The DNA fragment was ligated into the pDK6 vector to generate pDK6-hdpA. pDK6-hdpA was transformed into *Kp* Δ*tpiA* to generate *Kp* Δ*tpiA-hdpA*. Other strains with expression of *hdpA* were constructed in the same way as *Kp* Δ*tpiA-hdpA*.

### Medium and culture conditions

The fermentation medium contained 25 g/L glucose, 1.5 g/L yeast extract, 4 g/L (NH_4_)_2_SO_4_, 0.69 g/L K_2_HPO_4_·3H_2_O, 0.25 g/L KH_2_PO_4_, 0.2 g/L MgSO_4_, 0.05 g/L FeSO_4_·7H_2_O and 1 mL of trace element solution. One liter of trace element solution contained 200 mg CoCl_2_·6H_2_O, 100 mg MnSO_4_·4H_2_O, 70 mg ZnCl_2_, 60 mg H_3_BO_3_, 35 mg Na_2_MoO_4_·2H_2_O, 29.28 mg CuSO_4_·5H_2_O, 25 mg NiCl_2_·6H_2_O and 0.9 mL 37% HCl.

*K. pneumoniae* strains were inoculated in 250 ml flasks containing 50 ml medium and incubated on a rotary shaker at 37 °C and 120 rpm for 1 day. All experiments were done in triplicate, and data are expressed as the mean ± standard error.

The inhibitory effect of DHA on cell growth was determined in flask culture. Wild type *K. pneumoniae* were inoculated in 250 ml flasks containing 50 ml LB medium, and DHA was added in the medium to a obtain a range of different concentrations.

Fermentation parameters such as culture pH and oxygen supplementation optimization experiments were performed in batch bioreactor fermentation systems. For the seed culture, 250 mL flasks containing 50 mL of LB medium were incubated in a rotary shaker at 37 °C and 200 rpm overnight. Seed culture was inoculated into 5-L tank bioreactor (BIOSTAT-A plus, Sartorius) with a working volume of 3 L and the air flow rate was 2 L/min. The culture pH was automatically controlled by 10 mol/L NaOH addition. For fed batch culture, glucose was fed when its concentration in the broth was dropped to 5 g/L.

### Analytical methods

The biomass yield at set time intervals was determined by optical density (OD600) with a spectrophotometer. Cell dry weight (CDW) per liter of fermentation broth was calculated from OD values using the following formula: CDW (g/L) = 0.387 × OD + 0.334. Chemical compounds in the broth were quantified by a Shimadzu 20AVP high performance liquid chromatography system (HPLC) equipped with a RID-20A refractive index detector. An Aminex HPX-87H column (300 × 7.8 mm) (Bio-Rad, USA) was used and the column temperature was set at 65 ºC. The mobile phase was 0.005 mol/L H_2_SO_4_ solution with a flow rate of 0.8 ml/min.

The retention times of glycerol and DHA for HPLC were the same, thus gas chromatography (GC) was used to detect the levels of glycerol and DHA. A gas chromatography system (Shimadzu GC 2010) equipped with a flame ionization detector and a HP-FFAP column (30 m × 0.25 mm), with nitrogen as the carrier gas was used. The injector and detector were maintained at 250 °C and 280 °C, respectively. The column temperature initially started at 120 °C and was maintained for 1 min, then increased to 230 °C at a rate of 20 °C /min, then maintained at this temperature for 1.3 min.

Carbon balance was calculated according to the method described previous [5]. The elemental composition of *K. pneumoniae* was assumed to be CH_1.73_O_0.43_N_0.24_ [30]. The conversion ratio of DHA and glycerol from glucose was calculated as percentage of the produced DHA and glycerol (molar) divided by the consumed glucose (molar).

## Supplementary Information

**Additional file 1: Table S1. **Primers. **Figure S1.** Growth and product formation of *Kp *Δ*ptiA-*Δ*DHAK-hdpA* in shake flasks with different carbon sources. Data points are the average of n = 3; error bars represent standard error about the mean. **Figure S2.** Inhibition of the growth of *K. pneumoniae *by DHA. *K. pneumoniae *was grown in shake flasks with DHA added in the LB medium. Data points are the average of n = 3; error bars represent standard error about the mean.
