# Supplementary material for: Redirection of the central metabolism of Klebsiella pneumoniae towards dihydroxyacetone production
Source: Microb Cell Fact. 2021 Jun 29;20:123. doi: 10.1186/s12934-021-01608-0 (PMC8243499; doi:10.1186/s12934-021-01608-0)
Supplement: Supplementary file 1 — Additional file 1: Table S1. Primers. Figure S1. Growth and product formation of Kp ΔptiA-ΔDHAK-hdpA in shake flasks with different carbon sources. Data points are the average of n = 3; error bars represent standard error about the mean. Figure S2. Inhibition of the growth of K. pneumoniae by DHA. K. pneumoniae was grown in shake flasks with DHA added in the LB medium. Data points are the average of n = 3; error bars represent standard error about the mean. [file 12934_2021_1608_MOESM1_ESM.docx]

Table S1 Primers

| Primer name | Sequence (5’-3’) | |
| --- | --- | --- |
| tpiA-up-s | | CCCGGGGATCCTCTAGAGATCCGGCGCGCCGAGCTTTG |
| tpiA-up-a | | GCAGCTCCAGCCTACATTTAATTCTCCACGCTACTTAAGCG |
| tpiA-FRT-s | TAAATGTAGGCTGGAGCTGCTTCG | |
| tpiA-FRT-a | AAAAGCGGAATTCCGGGGATCCGTCGA | |
| tpiA-down-s | ATCCCCGGAATTCCGCTTTTCCCGGCGGC | |
| tpiA-down-a | CATGCCTGCAGGTCGACGATTAACGCTGCTGCCGCGGG | |
| mgsA- up-s | CCCGGGGATCCTCTAGAGATCTTTGGCGAAGGGGACTGC | |
| mgsA- up-a | AGCCTACATCGAACCGCGATCACGGC | |
| mgsA-FRT-s | ATCGCGGTTCGATGTAGGCTGGAGCTGCTTCG | |
| mgsA-FRT-a | GAAAATATTCCGGGGATCCGTCGA | |
| mgsA-down-s | CGGATCCCCGGAATATTTTCATTCCGCATTTTGACC | |
| mgsA-down-a | CATGCCTGCAGGTCGACGATAGGATAAAAAAACCCGCTAATGC | |
| gldA- up-s | CCCGGGGATCCTCTAGAGATCGGAGATGTACGCCGCCG | |
| gldA- up-a | CAGCCTACATCCAGAGCAAAACAAAACACCC | |
| gldA-FRT-s | TTTGCTCTGGATGTAGGCTGGAGCTGCTTCG | |
| gldA-FRT-a | AGGAATCGAAATTCCGGGGATCCGTCGA | |
| gldA-down-s | TCCCCGGAATTTCGATTCCTCCTCGATGACC | |
| gldA-down-a | CATGCCTGCAGGTCGACGATTGGGATGTACTGCCGCGC | |
| dhaD-up-s | CCCGGGGATCCTCTAGAGATTCAGACAGCAAAGCGAGATAGAGC | |
| dhaD-up-a | CTACATTCGCGGTGGCTAAACCG | |
| dhaD-FRT-s | TTTAGCCACCGCGAATGTAGGCTGGAGCTGCTTCG | |
| dhaD-FRT-a | GTGAAAGGAATTCCGGGGATCCGTCGA | |
| dhaD-down-s | ATCCCCGGAATTCCTTTCACCCTCAAATAAGTGC | |
| dhaD-down-a | CATGCCTGCAGGTCGACGATTCCGTTGGAGAAGGTTCGATG | |
| gapC-up-s | CCCGGGGATCCTCTAGAGATATTTACGATCGCTTCGTCAACC | |
| gapC-up-a | AGCTCCAGCCTACAGTGAACGGCGGACAGCCT | |
| gapC-FRT-s | GTTCACTGTAGGCTGGAGCTGCTTCG | |
| gapC-FRT-a | GAGAATATCCATTCCGGGGATCCGTCGA | |
| gapC-down-s | TCCCCGGAATGGATATTCTCCGGTTGGTAAAACG | |
| gapC-down-a | CATGCCTGCAGGTCGACGATCTGCAGGGATCTCGCCAGC | |
| gapA-up-s | CCCGGGGATCCTCTAGAGATTCACCGCCGCGATATCGC | |
| gapA-up-a | CCAGCCTACAGTTGAGATGAAAAAGTAATCTGTAAGAGC | |
| gapA -FRT-s | TCATCTCAACTGTAGGCTGGAGCTGCTTCG | |
| gapA -FRT-a | GGTGGAATATATTCCGGGGATCCGTCGA | |
| gapA-down-s | TCCCCGGAATATATTCCACCAGCTATTTGTTAGTGAA | |
| gapA-down-a | CATGCCTGCAGGTCGACGATATGCCGTGGGAGCTGTCG | |
| hdpA-s | TTCACACAGGAAACAGAATTCATGACAGTGAACATTTCATATCTGACC | |
| hdpA-a | TCCGCCAAAACAGCCAAGCTTCTAGTCAGTGAACTGCTGCTCATCT | |
| pDK6-s | CGCTACGGCGTTTCACTTC | |
| pDK6-a | CCAATACGCAAACCGCCTC | |
| pMD18-T-s | ATCGTCGACCTGCAGGCA | |
| pMD18-T-a | ATCTCTAGAGGATCCCCGGGT | |

Figure S1


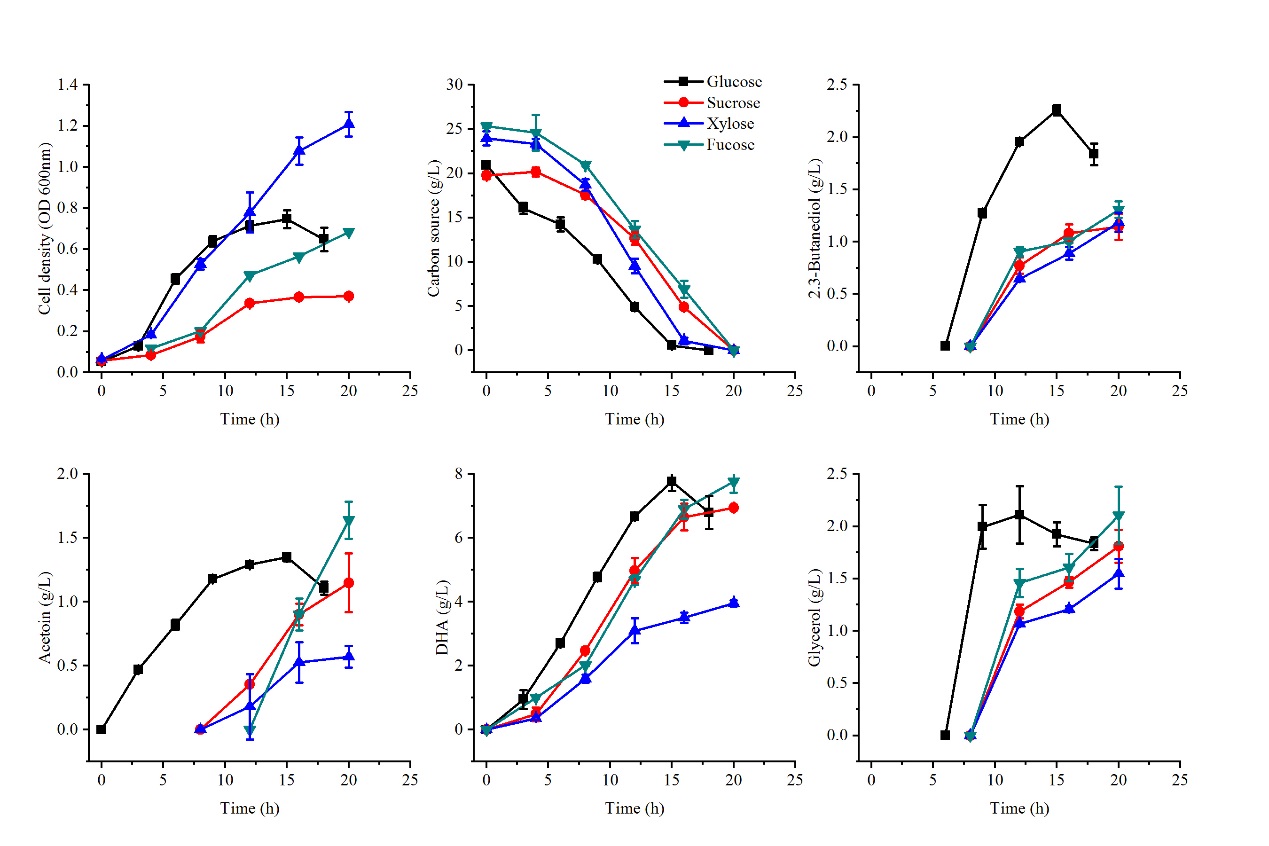


Figure S1. Growth and product formation of *Kp* Δ*ptiA-*Δ*DHAK-hdpA* in shake flasks with different carbon sources. Data points are the average of n = 3; error bars represent standard error about the mean.

Figure S2


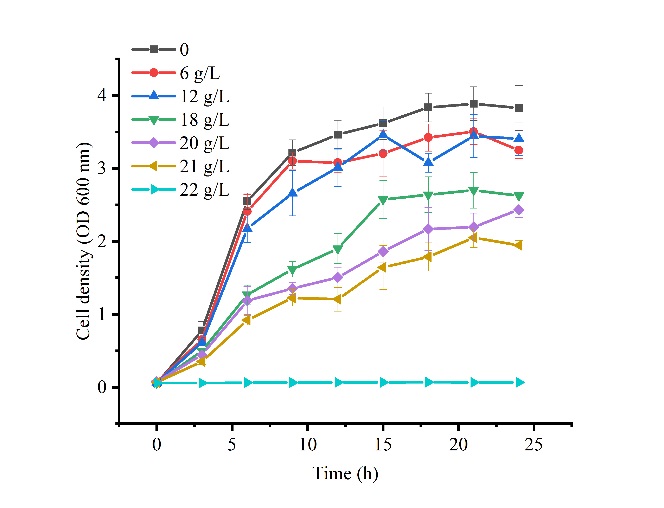


Figure S2. Inhibition of the growth of *K. pneumoniae* by DHA. *K. pneumoniae* was grown in shake flasks with DHA added in the LB medium. Data points are the average of n = 3; error bars represent standard error about the mean
